# Long‐Term Recurrence‐Free Survival After Surgery and Adjuvant Furmonertinib in a Patient With EGFR‐Mutated Pulmonary Sarcomatoid Carcinoma: A Case Report

**DOI:** 10.1002/cnr2.70729

**Published:** 2026-11-12

**Authors:** Minlian Nong, Xiang Tan

**Affiliations:** ^1^ Department of Geriatrics Respiratory Medicine The First Affiliated Hospital of Guangxi Medical University Nanning China; ^2^ Department of Thoracic Surgery The First Affiliated Hospital of Guangxi Medical University Nanning China

**Keywords:** EGFR mutation, molecular targeted therapy, pulmonary sarcomatoid carcinoma, surgery

## Abstract

**Background:**

Pulmonary sarcomatoid carcinoma (PSC) is an extremely rare subtype of non‐small cell lung cancer (NSCLC), which generally shows limited response to chemoradiotherapy and confers poor overall survival (OS). Recent progress in molecular targeted therapy has provided potential therapeutic strategies for this disease. Herein, we report a rare case of PSC harboring concurrent EGFR L858R and PIK3CA exon 10 mutations. The patient underwent radical surgical resection followed by adjuvant third‐generation epidermal growth factor receptor tyrosine kinase inhibitor (EGFR‐TKI) targeted therapy, and sustained long‐term disease‐free survival (DFS) was observed during clinical follow‐up. Although this case showed a favorable outcome, causality cannot be established from a single case report. Whether adjuvant furmonertinib contributed to the long‐term DFS remains uncertain and requires further investigation.

**Case:**

A middle‐aged female presented with cough, expectoration, and hemoptysis. Percutaneous lung biopsy confirmed Stage IIIA pulmonary sarcomatoid carcinoma, and radical surgical resection was subsequently performed. Postoperative next‐generation sequencing identified concomitant EGFR L858R and PIK3CA exon 10 mutations. The patient received adjuvant furmonertinib as postoperative targeted therapy. No tumor recurrence was detected throughout follow‐up, with a documented DFS of 48 months.

**Conclusion:**

Surgical resection remains the primary treatment for patients with early‐stage PSC, and postoperative molecular testing is critical to identify targetable mutations. This patient obtained durable DFS after radical resection combined with adjuvant third‐generation EGFR‐TKI targeted therapy. Additional large‐scale studies are needed to confirm the efficacy of adjuvant EGFR‐TKIs in PSC patients with dual EGFR/PIK3CA mutations.

## 1 Introduction

Pulmonary sarcomatoid carcinoma (PSC) is a rare subtype of non‐small cell lung cancer (NSCLC), accounting for less than 1% of primary lung cancers [1]. It predominantly affects elderly male patients with a smoking history. Originating from epithelial tissues, PSC arises through epithelial‐mesenchymal transition, thereby exhibiting dual characteristics of epithelial‐derived and mesenchymal‐derived tumors—manifested as strong vascular invasiveness, high distant metastasis rate, high malignancy, and poor prognosis. According to the 2015 World Health Organization (WHO) classification of lung tumors, PSC is categorized into five subtypes: pleomorphic carcinoma, spindle cell carcinoma, giant cell carcinoma, pulmonary blastoma, and carcinosarcoma [2].

Currently, there is no standard treatment for PSC, and management mainly refers to the principles for other NSCLC subtypes. For early‐stage disease, comprehensive treatment centered on surgery is preferred. For advanced‐stage patients, traditional radiotherapy and chemotherapy were historically the mainstay, but PSC responds poorly to these modalities, resulting in worse prognosis and higher mortality compared to other NSCLC subtypes [3]. With the application of molecular targeted therapy, immunotherapy, and anti‐angiogenic drugs in oncology, new treatment options have emerged for PSC, though supporting data from large‐scale studies remain limited. To date, molecular targeted therapy for PSC has been largely limited to case reports, predominantly focusing on MET mutations [4, 5]. EGFR mutations are rare in PSC patients, and most of them are rare site mutations. PIK3CA mutations are associated with resistance to epidermal growth factor receptor tyrosine kinase inhibitors (EGFR‐TKIs), poor immune response, and adverse prognosis in NSCLC patients. This article reports a case of PSC with concurrent EGFR L858R and PIK3CA mutations that achieved favorable prognosis with surgery combined with molecular targeted therapy.

## 2 Case Report

A 57‐year‐old female patient presented to our hospital (The First Affiliated Hospital of Guangxi Medical University) in February 2022 with a 3‐month history of cough and expectoration, accompanied by hemoptysis for more than 20 days. She denied a history of smoking or family history of malignancy. Three months prior to admission, she developed a cough with sputum production without any other accompanying symptoms. She received antitussive treatment at a community hospital without further evaluation. Twenty days prior to admission, she developed hemoptysis with bright red blood streaks. Community hospital chest radiography suggested a right lower lobe lung abscess. She was transferred to our hospital for further evaluation. The results of the complete blood count (CBC) were unremarkable, with low infection markers. And tumor marker test results were as follows: carbohydrate antigen 125 (CA125) 48.70 U/mL (reference range: 0.00–35.00 U/mL), neuron‐specific enolase (NSE) 23.29 ng/mL (reference range: 0–16.3 ng/mL), serum ferritin 208.10 ng/mL (reference range: 4.63–204 ng/mL). Cytokeratin 19 fragment and carcinoembryonic antigen (CEA) levels were within the normal reference range. Chest CT plain scan showed a mass‐like high‐density lesion in the right lower lobe, measuring approximately 7.8 × 6.5 × 7.7 cm, with air‐fluid levels. Imaging suggested a possible right lower lobe lung abscess with inflammation. But pathogen gene sequencing of bronchoalveolar lavage fluid (BALF) showed no meaningful positive results. Therefore, contrast‐enhanced chest CT was further conducted. The enhanced scan of the right lower lung lesion showed heterogeneous mild enhancement, with eccentric irregular cavities inside. Bronchi and blood vessels were seen running along the edge of the lesion, and the bronchus in the dorsal segment of the right lower lobe was narrowed; patchy increased density lesions were observed around the lesion (Figure 1A1,A2). Findings were suspicious for a tumor of the right lower lung. Ultrasound‐guided lung biopsy was performed, and histopathological examination with immunohistochemistry confirmed pulmonary sarcomatoid carcinoma. Further evaluations including lymph node ultrasound, abdominal ultrasound, whole‐body bone scintigraphy, and cranial CT showed no metastases. The patient was clinically staged as Stage IIIA (T4N0M0). Imaging examinations revealed no lymph node metastasis. After multidisciplinary team (MDT) discussion involving the departments of respiratory medicine, thoracic surgery, medical oncology, radiation oncology, radiology and anesthesiology, radical surgical resection was considered feasible to achieve complete tumor resection. The patient had a strong preference for surgical intervention and declined neoadjuvant chemotherapy. Therefore, the patient was subsequently transferred to the Department of Thoracic Surgery and underwent video‐assisted thoracoscopic radical resection of the right lung tumor in March 2022. Intraoperatively, the right middle and lower lobes were resected, along with a 9 × 9 × 4 cm mass. Lymph node dissection was performed for stations 2, 4, 7, 9, 10, 11, and 12, with a total of 22 lymph nodes harvested. Postoperative pathological examination confirmed no lymph node metastasis in all specimens. On histopathological examination, the tumor shows diffusely distributed atypical cells and cancer nests, containing such components as adenocarcinoma, squamous cell carcinoma, fibrosarcoma, and chondrosarcoma‐like elements, along with extensive necrosis. The tumor is composed of approximately 90% high‐grade pleomorphic sarcoma, 10% moderately differentiated adenocarcinoma, and a small amount of keratinizing squamous cell carcinoma. Immunophenotypic analysis reveals CK, CK7, and TTF‐1 (epithelium+); Vimentin (mesenchyme+), S‐100(−), CD34(−), CD68 (scattered+), Desmin (scattered+), SMA (partial+), Caldesmon (+), Actin (−), Myogenin (−); and a Ki‐67 index of 30% (Figure 2A–L). These findings support the diagnosis of PSC. Genetic testing revealed EGFR L858R mutation, PIK3CA exon 10 mutation, and PD‐L1 expression of 1%. Given that the patient was diagnosed with Stage IIIA malignant tumor, harbored an EGFR L858R activating mutation, and exhibited low PD‐L1 expression, and the patient declined postoperative radiotherapy, chemotherapy, and immunotherapy. With the patient's informed consent, furmonertinib 80 mg was administered once daily as adjuvant targeted therapy starting from April 2022. The patient received regular follow‐up with serial re‐examinations of tumor markers and imaging studies. To date, no signs of recurrence have been identified, and no adverse events related to EGFR‐TKI treatment have occurred (Figures 1B–F and 3). At the last follow‐up on March 4th, 2026, the patient underwent a battery of examinations, including chest CT (Figure 1F1,F2), cranial CT, lymph node and abdominal ultrasound, and whole‐body bone scintigraphy. The clinical course and treatment timeline of this patient are summarized in Table 1. As of now, the patient's condition remains stable, with a disease‐free survival (DFS) of 48 months.

**FIGURE 1:**
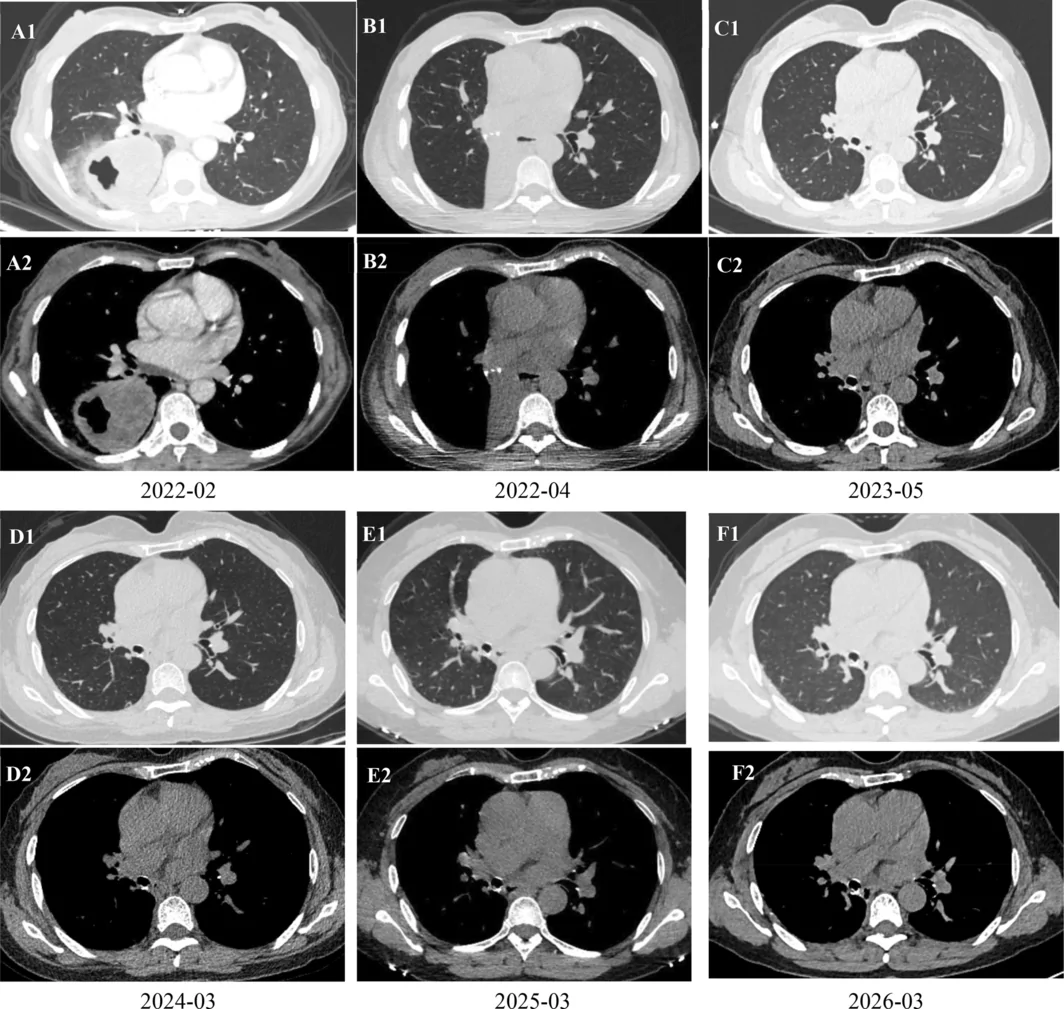
Schematic of chest CT in patient's treatment history (2022–2026). A1 + A2 baseline (before surgery), B1 + B2 (3 weeks after surgery, 2 weeks before targeted therapy), C1 + C2 (14 months after surgery,13 months on targeted therapy), D1 + D2 (24 months after surgery,23 months on targeted therapy), E1 + E2 (36 months after surgery,35 months on targeted therapy), F1 + F2 (latest follow‐up:48 months after surgery,47 months on targeted therapy).

**FIGURE 2:**
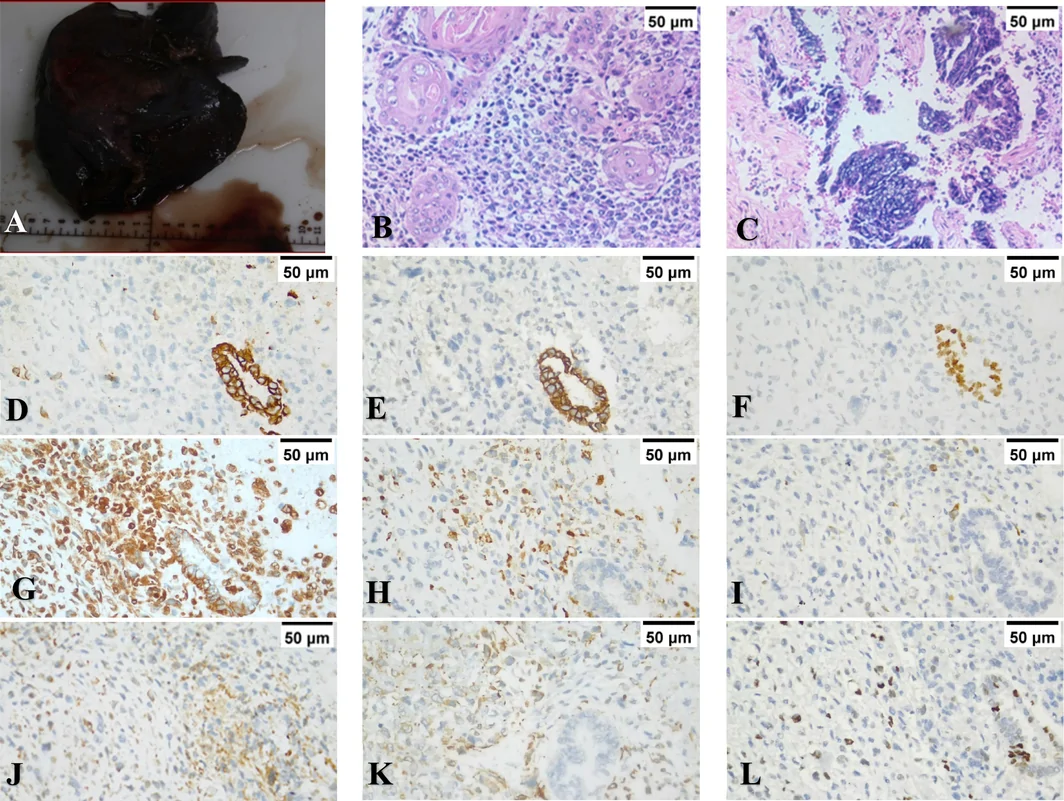
(A) 2022–03 Patient's Intraoperative Tumor Resection Image. (B, C) Hematoxylin–eosin (HE) staining, showing diffusely distributed atypical cells and tumor nest formation. (D–L) Immunohistochemical staining. (D) CK (epithelium +); (E) CK7 (epithelium +); (F) TTF‐1 (epithelium+); (G) Vimentin (mesenchyme+); (H) CD68 (scattered+); (I) Desmin (scattered+); (J) SMA (partial+); (K) Caldesmon (+); (L) Ki‐67 (30%).

**FIGURE 3:**
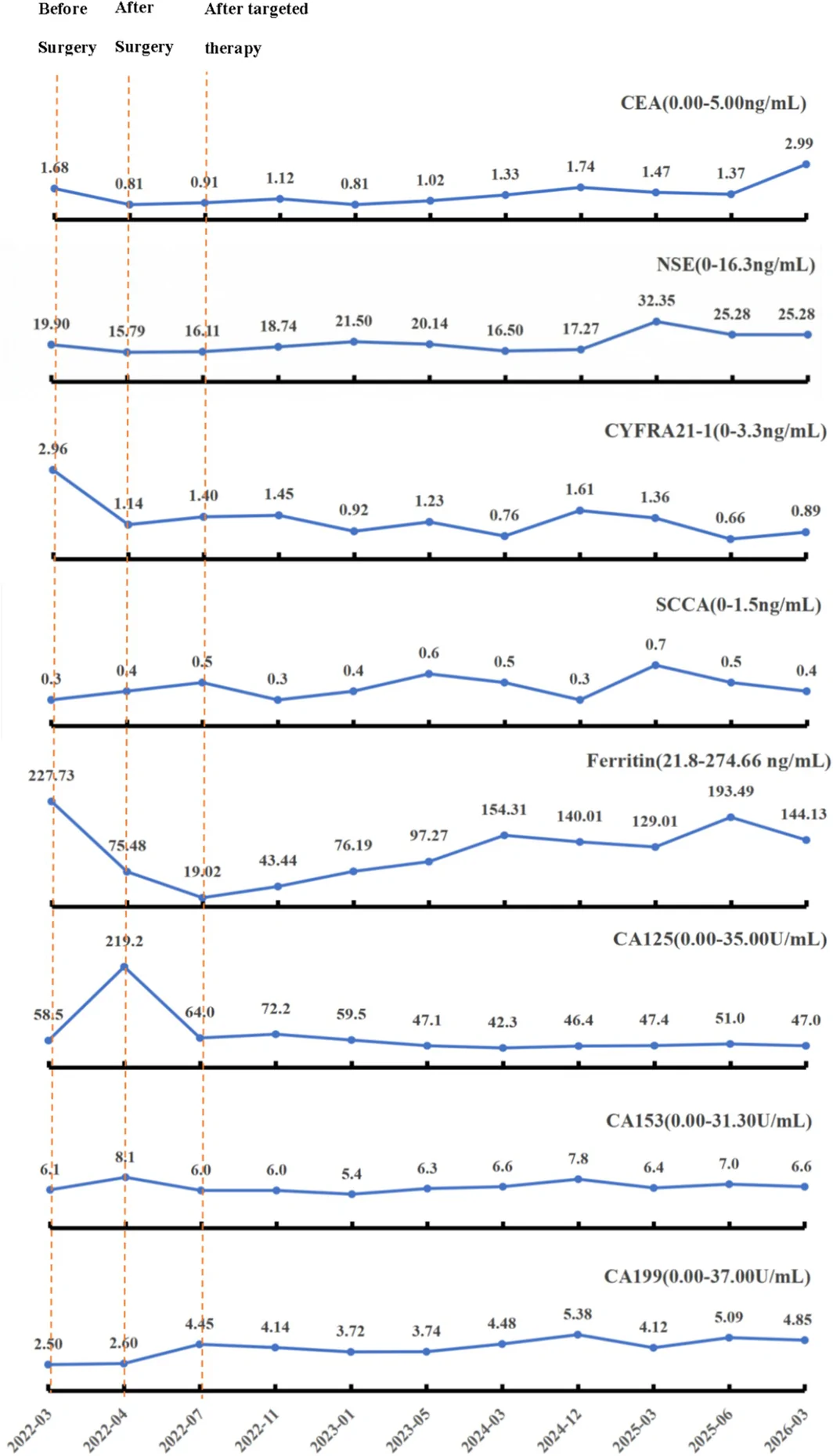
Dynamic graph of tumor marker changes in patient's treatment history (2022–2026).

**TABLE 1:** Clinical course and treatment timeline.

Date (month/year)	Clinical event/intervention	Details and findings
November 2021	Symptom onset	Initial presentation of cough and expectoration. Received empirical antitussive therapy at a community hospital without imaging.
February 2022	Initial presentation and diagnosis	Admitted to our hospital due to hemoptysis. Chest CT revealed a mass in the right lower lobe (7.8 × 6.5 × 7.7 cm) with air‐fluid levels. Ultrasound‐guided lung biopsy performed.
February 2022	Pathological confirmation	Histopathology and IHC confirmed PSC. Clinical staging: IIIA (T4N0M0).
March 2022	Surgical resection	Underwent Video‐assisted thoracoscopic radical resection (Right middle and lower lobectomy). Lymph node dissection (2,4,7,9,10,11,12 stations; 22 nodes total). Post‐op pathology: No lymph node metastasis; Tumor composed of 90% sarcoma +10% adenocarcinoma.
March 2022	Molecular testing	Genetic testing revealed *EGFR* L858R mutation, *PIK3CA* exon 10 mutation, and PD‐L1 expression 1%. Patient declined adjuvant chemo/radiotherapy/immunotherapy.
April 2022	Initiation of adjuvant therapy	Started targeted therapy with furmonertinib 80 mg once daily. as first‐line adjuvant treatment.
April 2022–Present	Maintenance therapy and monitoring	Regular follow‐ups with serial tumor marker tests and imaging (chest CT, cranial CT, ultrasound, bone scintigraphy). No AEs reported.
Latest Follow‐up Assessment	Latest follow‐up assessment	Latest comprehensive imaging battery showed no signs of recurrence or metastasis. Patient remains in stable condition.

## 3 Discussion

Current management of PSC primarily refers to other NSCLC subtypes. Surgical resection remains the first‐line treatment for patients with early‐stage PSC and can improve their overall prognosis [6, 7, 8, 9, 10]. For patients with intermediate and advanced‐stage disease, multimodal comprehensive regimens including surgical resection, radiotherapy, chemotherapy, and targeted therapy can be adopted. Nevertheless, there is no unified consensus regarding the clinical value of adjuvant chemoradiotherapy, and the efficacy of radiotherapy in PSC treatment remains unclear [11]. However, neoadjuvant or adjuvant chemotherapy has been shown to effectively prolong progression‐free survival (PFS) and overall survival (OS) in some cases [12]. In recent years, molecular targeted therapy, immunotherapy, and anti‐angiogenic therapy have demonstrated varying efficacy in PSC, but data are limited. Data on genetic mutations in PSC are uncommon. Known mutations include KRAS, TP53, ALK, MET, EGFR, PIK3CA, BRAF, HER2, RET, CDKN2A, PTEN, NOTCH, STK11, and RB1, with KRAS and TP53 being the most common. There are no effective drugs for these mutations in clinical practice. Most common driver mutations in NSCLC in lung adenocarcinoma or squamous cell carcinoma are rare in PSC [13, 14], limiting the role of molecular targeted therapy in PSC. Additionally, the relationship between PSC mutations and those in other NSCLC subtypes, as well as their correlation with race, gender, and smoking status, remains unclear and requires further investigation. EGFR mutations, common in Asian NSCLC patients, occur in 0%–28% of PSC cases [14, 15, 16]. Due to small sample sizes and variations in race and region, reported rates vary widely. In addition, existing data indicate that EGFR mutations in PSC predominantly involve rare loci, while common mutations in exons 19, 20, and 21 are rare. With the development of third‐generation TKIs, an increasing number of cases have reported favorable outcomes in PSC [17, 18, 19]. PIK3CA mutations occur in 5%–8% of NSCLC patients, predominantly in lung adenocarcinoma, often coexist with EGFR, TP53, or KRAS mutations [20]. Studies have linked PIK3CA mutations to poor prognosis in NSCLC, particularly in patients with concurrent EGFR and PIK3CA mutations, who exhibit shorter PFS and OS [21]. A retrospective cohort study found that PIK3CA mutations correlate with poor postoperative prognosis in PSC: three patients with recurrent PSC harboring pathogenic PIK3CA mutations survived less than 2 years despite chemotherapy or immunotherapy [22]. This may be attributed to PIK3CA mutations promoting tumor progression, inducing EGFR‐TKI resistance, and impairing immune responses [23]. PI3K inhibitors, which have demonstrated benefits in breast cancer, lack sufficient clinical data in NSCLC.

The patient, a middle‐aged female with Stage IIIA PSC, harbored EGFR L858R and PIK3CA exon 10 mutations, with PD‐L1 expression of 1%. No relevant case reports were retrieved from PubMed with the keywords pulmonary sarcomatoid carcinoma, EGFR and PIK3CA. In this case, despite the patient carrying the PIK3CA mutation—a gene associated with poor prognosis—she also presents with the EGFR L858R mutation, which is frequently observed in NSCLC cases. The NCCN guidelines recommend adjuvant treatment with osimertinib, a third‐generation EGFR TKI, for patients with Stage IIIA NSCLC harboring EGFR 19del or L858R mutations following radical resection [24, 25]. While EGFR‐TKI agents have demonstrated well‐established efficacy, particularly in lung adenocarcinoma patients, no such findings have been documented in PSC populations to date. Furmonertinib, a third‐generation TKI independently developed in China, has been shown to significantly extend PFS in Chinese patients with advanced EGFR‐mutant NSCLC. It is also effective in NSCLC patients with brain metastasis or EGFR T790M mutation (a known resistance mechanism), with a favorable safety profile even at high doses [26, 27, 28]. In addition, one case report described marked clinical benefits achieved with furmonertinib as fourth‐line therapy for advanced lung adenocarcinoma harboring concurrent EGFR exon 20 insertion mutations and PIK3CA mutations. This suggests that furmonertinib may yield prominent clinical benefits in patients with NSCLC with complex resistance mechanisms, including those with resistance related to aberrant PIK3CA/mTOR pathway signaling [29]. The patient presented with Stage IIIA PSC and achieved complete tumor resection. Postoperative molecular testing confirmed EGFR mutation positivity, and the patient declined postoperative adjuvant radiotherapy, chemotherapy and immunotherapy. Considering the established efficacy, safety and cost‐effectiveness of furmonertinib in Chinese NSCLC populations, postoperative targeted therapy with furmonertinib 80 mg once daily was administered. The median PFS is only approximately 5–9 months under systemic therapy. Even for resected Stage III PSC, the median DFS is generally unsatisfactory, and relapse is common within the first 2 years after surgery [30, 31]. Notably, our patient achieved a DFS of 48 months after radical resection and adjuvant third‐generation EGFR‐TKI targeted therapy, which is markedly longer than most reported outcomes for PSC, especially considering the rarity of EGFR‐mutated PSC. This favorable long‐term disease‐free survival may be attributed to multiple factors, including complete radical resection, adjuvant EGFR‐targeted therapy, and favorable intrinsic tumor biology.

Given the high propensity of PSC for vascular invasion, anti‐angiogenic drugs have been increasingly studied in PSC. Reports suggest that single‐agent or chemotherapy‐combined anti‐angiogenic therapy may modestly improve OS and PFS, but data are limited and require validation. Bevacizumab, which is beneficial in advanced lung adenocarcinoma, has also shown potential in PSC when combined with chemotherapy [32]. Anlotinib has been reported to benefit individual PSC patients [33, 34]. Bevacizumab and anlotinib may represent treatment options for advanced PSC, but their mechanisms of action in PSC require further exploration.

PSC typically exhibits higher PD‐L1 expression compared to other NSCLC subtypes, particularly in elderly male smokers, differing from common NSCLC [35, 36]. Immunotherapy research in PSC is growing but lacks large‐scale data. Notably, some PD‐L1‐positive PSC patients fail to respond to immunotherapy, suggesting a disconnect between PD‐L1 expression and efficacy [36, 37], possibly due to unique immune escape mechanisms in PSC [35, 38]. PIK3CA mutations activate the PI3K/AKT/mTOR pathway, which regulates the tumor immune microenvironment, promotes tumorigenesis, and upregulates PD‐L1 expression, leading to resistance to immune checkpoint inhibitors. Thus, PSC patients with PIK3CA mutations may have poor responses to immunotherapy [39]. In this case, the patient was a middle‐aged non‐smoking female with low PD‐L1 expression of 1% and PIK3CA exon 10 mutation, making the benefit of postoperative immunotherapy uncertain. Given the confirmed EGFR exon 21 mutation, immunotherapy was not prioritized. The effectiveness of immunotherapy for PSC patients remains to be further explored.

Although PSC responds poorly to traditional radiotherapy and chemotherapy, molecular targeted therapy, immunotherapy, and anti‐angiogenic drugs have shown potential benefits, offering hope for improved prognosis. Surgical resection should be prioritized for PSC patients meeting surgical indications. Genetic testing is strongly recommended regardless of disease stage to identify actionable molecular targets for targeted therapy. Herein, we report one patient who achieved sustained disease‐free survival after surgery combined with adjuvant third‐generation EGFR‐TKI therapy. However, as this is merely a single case report lacking comparative control data, further evidence from additional studies is required to validate these observations.

## Author Contributions

**Minlian Nong:** conceptualization, methodology, data curation, investigation, writing – original draft. **Xiang Tan:** conceptualization, methodology, investigation, funding acquisition, writing – review and editing.

## Funding

Funding for this work was provided by the Guangxi Science and Technology Program (No. GK2025GXNSFAA069077), and the Self‐funded Scientific Research Project of the Health Commission of Guangxi Zhuang Autonomous Region (No. Z‐A20220398).

## Ethics Statement

This study comprises a case report and literature review.

## Consent

The patient provided written informed consent to publish this case report. All identifying information has been de‐identified, and the manuscript complies with the CARE guidelines.

## Conflicts of Interest

The authors declare no conflicts of interest.

## Data Availability

The datasets used and/or analyzed during the current study are available from the corresponding author on reasonable request.
